# CXCR4 expression in glioblastoma tissue and the potential for PET imaging and treatment with [^68^Ga]Ga-Pentixafor /[^177^Lu]Lu-Pentixather

**DOI:** 10.1007/s00259-021-05196-4

**Published:** 2021-02-07

**Authors:** Sarah M. Jacobs, Pieter Wesseling, Bart de Keizer, Nelleke Tolboom, F. F. Tessa Ververs, Gerard C. Krijger, Bart A. Westerman, Tom J. Snijders, Pierre A. Robe, Anja G. van der Kolk

**Affiliations:** 1 Department of Radiology and Nuclear Medicine, University Medical Center Utrecht, Utrecht, the Netherlands; 2 Princess Máxima Center for Pediatric Oncology, Utrecht, the Netherlands; 3 Department of Pathology, Amsterdam University Medical Centers/VUmc, Amsterdam, the Netherlands; 4 Department of Clinical Pharmacy, University Medical Center Utrecht, Utrecht, the Netherlands; 5 UMC Utrecht Brain Center, Department of Neurology and Neurosurgery, University Medical Center Utrecht, Utrecht, the Netherlands; 6 Department of Radiology, Netherlands Cancer Institute/Antoni van Leeuwenhoek Hospital, Amsterdam, the Netherlands

**Keywords:** Glioblastoma, CXCR4, PET, [^68^Ga]Ga-Pentixafor, [^177^Lu]Lu-Pentixather, Molecular imaging

## Abstract

**Purpose:**

CXCR4 (over)expression is found in multiple human cancer types, while expression is low or absent in healthy tissue. In glioblastoma it is associated with a poor prognosis and more extensive infiltrative phenotype. CXCR4 can be targeted by the diagnostic PET agent [^68^Ga]Ga-Pentixafor and its therapeutic counterpart [^177^Lu]Lu-Pentixather. We aimed to investigate the expression of CXCR4 in glioblastoma tissue to further examine the potential of these PET agents.

**Methods:**

CXCR4 mRNA expression was examined using the R2 genomics platform. Glioblastoma tissue cores were stained for CXCR4. CXCR4 staining in tumor cells was scored. Stained tissue components (cytoplasm and/or nuclei of the tumor cells and blood vessels) were documented. Clinical characteristics and information on IDH and *MGMT* promoter methylation status were collected. Seven pilot patients with recurrent glioblastoma underwent [^68^Ga]Ga-Pentixafor PET; residual resected tissue was stained for CXCR4.

**Results:**

Two large mRNA datasets (*N* = 284; *N* = 540) were assesed. Of the 191 glioblastomas, 426 cores were analyzed using immunohistochemistry. Seventy-eight cores (23 tumors) were CXCR4 negative, while 18 cores (5 tumors) had both strong and extensive staining. The remaining 330 cores (163 tumors) showed a large inter- and intra-tumor variation for CXCR4 expression; also seen in the resected tissue of the seven pilot patients—not directly translatable to [^68^Ga]Ga-Pentixafor PET results. Both mRNA and immunohistochemical analysis showed CXCR4 negative normal brain tissue and no significant correlation between CXCR4 expression and IDH or *MGMT* status or survival.

**Conclusion:**

Using immunohistochemistry, high CXCR4 expression was found in a subset of glioblastomas as well as a large inter- and intra-tumor variation. Caution should be exercised in directly translating ex vivo CXCR4 expression to PET agent uptake. However, when high CXCR4 expression can be identified with [^68^Ga]Ga-Pentixafor, these patients might be good candidates for targeted radionuclide therapy with [^177^Lu]Lu-Pentixather in the future.

**Supplementary Information:**

The online version contains supplementary material available at 10.1007/s00259-021-05196-4.

## Introduction

Glioblastoma is the most common primary malignant brain tumor in adults, with a median survival of 15 months; only 5.6% of patients survive 5 years post-diagnosis despite aggressive treatment, consisting of a combination of salvaging surgery and temozolomide-based chemoradiotherapy [1]. Clear delineation of the tumor and subsequent treatment planning remains a challenge. Due to its high soft tissue contrast and tumor sensitivity, conventional magnetic resonance imaging (MRI) is the standard of diagnostic care for brain tumors. Conventional MRI sequences, however, lack tumor cell specificity because they are based on imaging the movement and interaction of water protons in tissues, which is relatively nonspecific for individual tissue types.

Molecular MRI techniques—such as MR spectroscopy and chemical exchange saturation transfer (CEST)—could improve tumor specificity of MRI by visualizing molecules that are specific to tumor metabolism, but these have not yet been fully integrated into standard clinical care [2, 3]. Positron emission tomography (PET) is similar to metabolic MRI in that it targets molecular characteristics of human tissues. In addition, by physically targeting certain tumor-specific molecules, a diagnostic PET agent could also serve as a therapeutic agent. In the last decades, several PET agents have shown their potential for brain tumor imaging [4]. Nonetheless, most of these agents, although more specific than conventional MRI, are still hampered by a relative low specificity for tumor cells [5].

The PET agent [^68^Ga]Ga-Pentixafor—with its associated therapeutic version, [^177^Lu]Lu-Pentixather—has been developed as a new potential agent for brain tumor imaging and targeted radionuclide therapy. [^68^Ga]Ga-Pentixafor attaches to the C-X-C chemokine receptor type 4 (CXCR4), a G protein coupled receptor for the ligands stromal-derived-factor 1 (SDF-1)—also known as C-X-C motif chemokine 12 (CXCL12)—and macrophage inhibitory factor (MIF). In healthy cells, in particular in bone marrow, CXCR4 plays an important role in cellular survival, proliferation, migration, and chemotaxis as well as angiogenesis. [6–9] (Over) expression of CXCR4 has been shown in several human cancer types, such as ovarian, prostate, and esophageal as well as malignant glioma [7, 10]. Increasing CXCR4 expression has been reported in astrocytomas with higher WHO grade [11, 12]. In glioblastoma patients it is associated with a poor prognosis and a more extensively infiltrative phenotype [13–18]. Compared with [^68^Ga]Ga-/[^177^Lu]Lu-PSMA, an already established theranostics agent that attaches to the prostate-specific membrane antigen (PSMA) present on the neovasculature of many tumor types [19], CXCR4 is present on both the neovasculature and the tumor cells. This brings an advantage to treatment with [^177^Lu]Lu-Pentixather, as it can be directed at tumor cells *and* neovasculature when CXCR4 is present.

A previous clinical pilot study using [^68^Ga]Ga-Pentixafor in glioblastoma patients showed promising results, with tumor uptake of the PET agent in 11/13 glioblastoma patients [20]. However, the small sample size of this clinical study prompted us to investigate the degree of expression of CXCR4 in glioblastoma tumor cells and healthy brain tissue in a larger population, to further examine the potential of [^68^Ga]Ga-Pentixafor and [^177^Lu]Lu-Pentixather as, respectively, diagnostic and therapeutic PET agents in glioblastomas.

## Materials and methods

This study comprised three parts: (1) a large database analysis for CXCR4 mRNA expression in different glioma grades and tissues, (2) ex vivo histopathological tissue assessment of CXCR4 expression in patients with glioblastoma using CXCR4 staining, and (3) direct correlation of in vivo [^68^Ga]Ga-Pentixafor binding with CXCR4 expression ex vivo in patients with suspicion of recurrent glioblastoma.

### CXCR4 mMRNA expression

As a first step, the R2 genomics platform [21] was used to asses mRNA expression of CXCR4 in glioma and glioblastoma tissue, to provide insight into the prevalence of CXCR4 (over)expression in a large patient sample. The R2 genomics platform is a free, publicly accesible web-based genomics analysis and visualization platform with access to a large variety of gene expression datasets. For the current study, two datasets were used: (1) the GEO dataset GSE16011 [22], which contains information on different WHO glioma and normal brain tissue, used for analysis of expression rates of CXCR4 mRNA in different glioma grades, and (2) the glioblastoma TCGA dataset [23], containing information on IDH status and *MGMT* promoter methylation in gliomas, used to assess possible correlations between CXCR4 mRNA expression and molecular tumor markers.

### Ex vivo CXCR4 staining

CXCR4 mRNA expression analysis provides indirect insight into expression of CXCR4 in or on a cell in a large patient sample. However, objectifying this expression *ex vivo* on tissue samples provides more concrete evidence of the presence of the PET-targeted molecule on the cell membrane. Therefore, four Tissue Microarrays (TMAs) created in 2014 for a different histopathological study and consisting of formalin-fixed, paraffin-embedded tissue samples (cores) of consecutive patients with a histopathological diagnosis of glioblastoma and operated between 2005 and 2014 were used and stained with anti-CXCR4 (mouse monoclonal, dilution 1:800; BioLegend, San Diego, CA, USA). Six liver samples and three kidney samples were used to validate the stain. A standard hematoxylin and eosin (HE) staining was available for all four TMAs to discriminate different cell types and other components when unclear on CXCR4-stained cores. Four samples of normal brain tissue taken from epilepsy surgery cases for clinical purposes were stained for CXCR4 to serve as a control group. Clinical characteristics (gender, date of birth, date of diagnosis, date of death or last seen alive, and histopathological diagnosis) as well as information on molecular tumor markers (isocitrate dehydrogenase (IDH) status and methyl guanine-deoxyribonucleic acid methyltransferase (*MGMT*) promoter methylation status) were collected from the electronic medical records. For the majority information on the IDH status was obtained by means of immunohistochemistry for IDH1R132H. Permission for this retrospective study was obtained from the Biobank Research Ethics committee of our institution based in Utrecht, the Netherlands; written informed consent was waived.

### TMA assessment

**Fig. 1:**
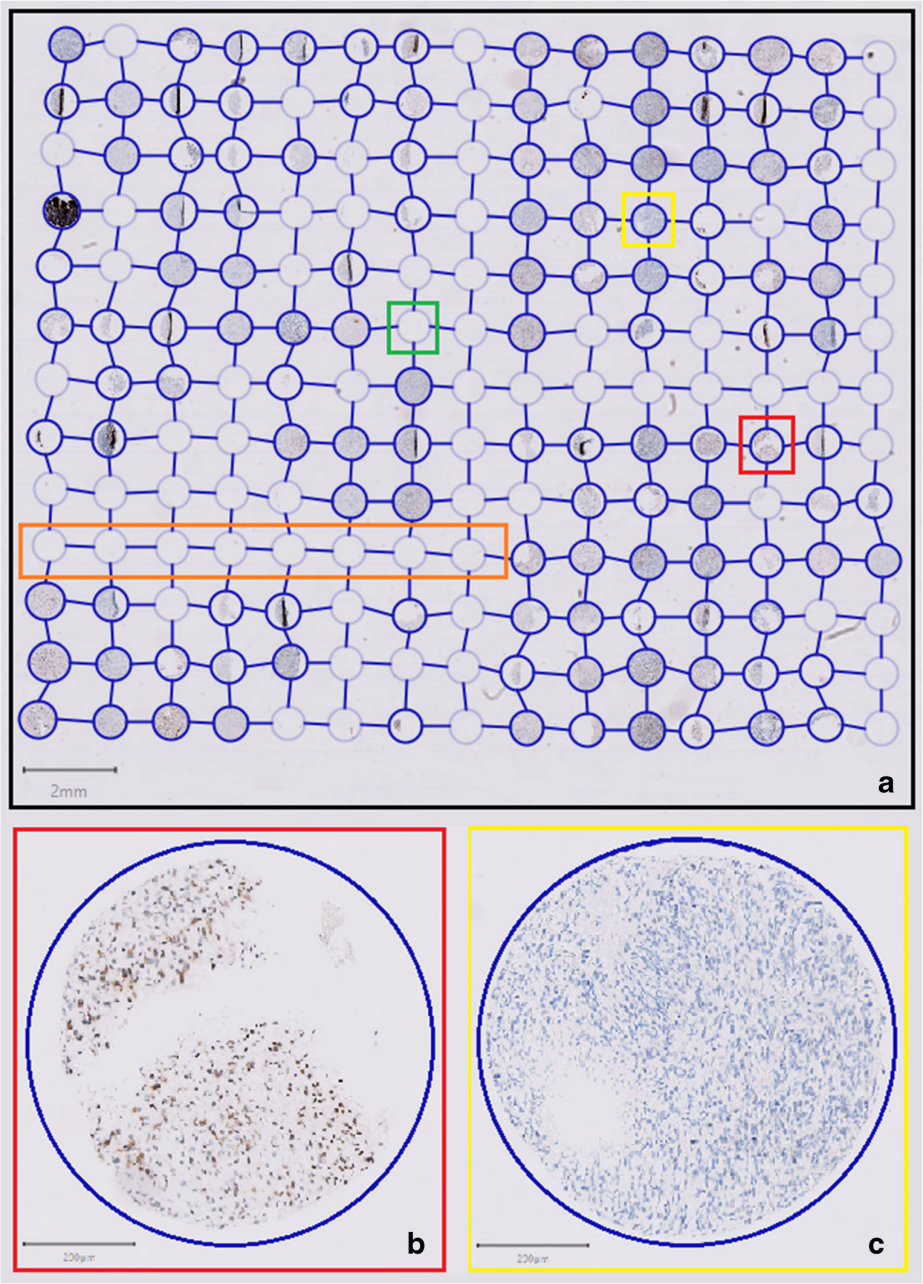
Example of a single tissue microarray (TMA) with CXCR4 stained glioblastoma tissue cores: (**a**) TMA grid in blue; green square indicating empty core due to missing tissue; orange rectangle indicating row of empty cores being part of the grid for orientation; yellow and red square representing CXCR4 positive versus CXCR4 negative glioblastoma tissue cores, higher magnification in, respectively, (**b**) and (**c**)

First, all TMAs were scanned for digital use. Second, a grid was developed for each TMA with the program QuPath (version 0.1.2), an open software platform for whole slide image analysis created at the Centre for Cancer Research & Cell Biology at Queen’s University Belfast, currently under development at Edinburgh University [24]. Labels of each core were added to the grid to create a look-up for each core and allowing the individual cores of the CXCR4 and HE staining to be compared one on one (Fig. 1). Subsequent scoring of the CXCR4-stained cores was performed by one researcher (SJ) who had been trained by an experienced neuropathologist (PW). In addition, 15 randomly chosen cores were scored by the same neuropathologist for interobserver agreement calculations. All cores were screened for positive (i.e. any) CXCR4 staining. Cores with established positive CXCR4 staining were then scored for intensity and extensiveness of staining of the tumor cells, using an arbitrary 4-point scale. Intensity was divided into no staining (-), subtle staining (+), moderate staining (++), and strong staining (+++). Extensiveness was defined as an estimated percentage of the area of the core showing CXCR4 staining and divided into 0% (negative), less than 25% (limited), 25–75% (partial), and 75% or higher (diffuse). In addition, the presence of different CXCR4-positive tissue components was documented, differentiating between blood vessels, cytoplasm, and/or nuclei of the tumor cells.

### In vivo-ex vivo correlation

In the final step, PET images from seven patients with suspicion of recurrent glioblastoma who underwent [^68^Ga]Ga-Pentixafor PET-CT as part of clinical care were retrospectively assessed and compared with CXCR4-stained sections of the surgically resected tissue of these patients. Similar to the TMA assessment, sections were scored for intensity of CXCR4 staining of the tumor cells throughout the section as well as the presence of stained tumor vessels. For each patient, one section was available; these sections were on average three times larger in size than the TMA cores.

[^68^Ga]Ga-Pentixafor was prepared in house and administered intravenously with an activity of 1.5 MBq/kg, as described earlier for [^68^Ga]Ga-PSMA [25]. Combined PET and CT images were acquired approximately 45 min after injection and consisted of a single bed position and acquisition time of 10 min, using a TruePoint Biograph mCT40 scanner (Siemens, Erlangen, Germany). PET was reconstructed according to the European Association of Nuclear Medicine recommendations with the following parameters: PET with time-of-flight and point spread function (TrueX) reconstruction, four iterations, 21 subsets, with a filter of 7.5 mm full width at half maximum [26]. For standardized uptake value (SUV) measurements, the lean body mass corrected values were used.

### Statistical analysis

Statistical analysis was performed by means of SPSS version 25 (IBM Analytics).

Comparison of expression rates of CXCR4 mRNA in different glioma grades and possible correlations between CXCR4 mRNA expression and molecular tumor markers were assessed using ANOVA.

Interobserver agreement for tumor cell and blood vessel staining was calculated with Cohen’s Kappa. Intensity and extensiveness scores per core were presented as descriptive statistics. To analyze possible correlations between CXCR4 staining and molecular tumor markers, the presence or absence of IDH status respectively *MGMT* methylation was assessed on a core level using Chi-Square (trend) or Fisher’s exact test (i.e., IDH mutant or IDH wild-type respectively *MGMT* methylated or *MGMT* unmethylated versus intensity score (4 categories) respectively extensiveness score (4 categories)).

Next, to increase clinical relevance, CXCR4 staining scores were combined on a core level, and cut-off values were arbitrarily defined to translate core scores to a *tumor level*. As an example, one of the cut-off values was defined as intensity “++” and extensiveness “25–75%”; in this case, all cores with an intensity score below “++” and/or an extensiveness score < 25% were defined as being CXCR4 “negative,” while the others were CXCR4 “positive.” These results were then translated to a tumor level, where a tumor with only “positive” cores would be “CXCR4 positive,” one with only negative cores “CXCR4 negative,” and a tumor with both positive and negative cores “CXCR4 mixed.”

Molecular tumor markers were again compared with CXCR4 staining—but now on a tumor level—using Chi-Square (trend) or Fisher’s exact test. In addition, Kaplan-Meier curves were used to assess differences in survival between patients with and without CXCR4-positive cores and patients with and without CXCR4-positive tumors.

Results from the in vivo-ex vivo comparison were described in detail for each patient individually. In addition means were calculated for the SUVmean of the tumor, bloodpool and background (defined as uptake in the white and gray matter of the controlateral hemisphere), and SUVmax of the tumor and background as well as (mean) tumor-to-background ratios (TBR) for the SUVmean and SUVmax of the tumor in comparison to the SUVmean of the background.

## Results

### CXCR4 mRNA expression

**Fig. 2:**
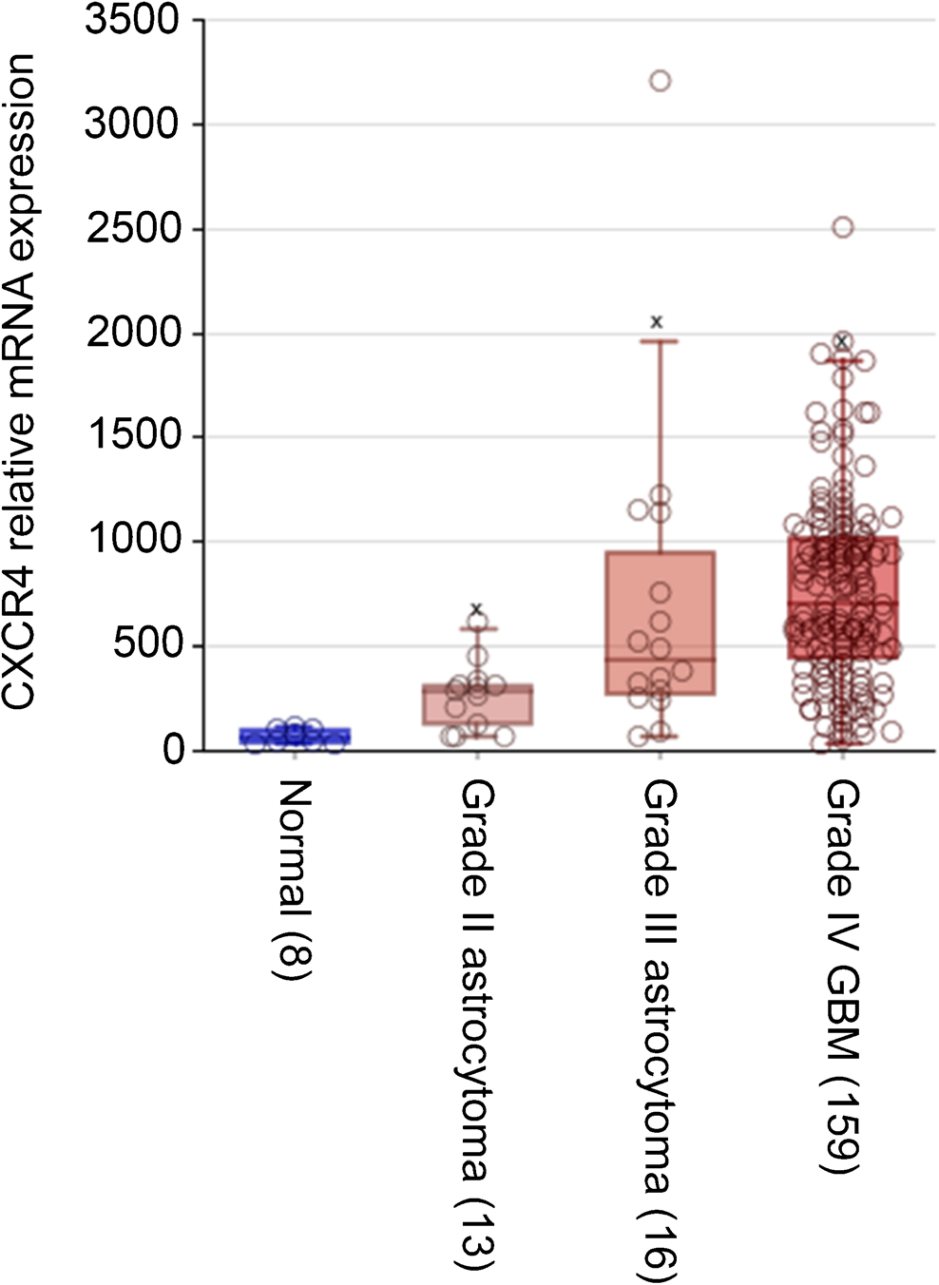
CXCR4 mRNA expression according to WHO glioma grade and in normal brain tissue determined with GEO dataset GSE16011 (*N* = 284), showing a relatively higher expression in high grade gliomas, with highest (very variable) expression in glioblastoma, while normal brain tissue did not show expression. Data are presented as box and whisker plots: boxes extend from the 25th to 75th percentile, with a black line at the median. Statistical significance was determined by ANOVA, *p* < 0.05

The correlation between CXCR4 mRNA expression and WHO glioma grade was determined with the use of GEO dataset GSE16011 (*N* = 284), and showed a relatively higher expression in high grade gliomas, whereas normal brain tissue was CXCR4 mRNA negative. Glioblastomas showed a large variation in CXCR4 mRNA expression and, however, were also the highest compared with lower grades (Fig. 2). Using the data from the glioblastoma TCGA dataset (*N* = 540), no significant correlation between CXCR4 mRNA expression in glioblastoma and IDH status or *MGMT* promoter methylation was found, nor between CXCR4 mRNA expression and survival.

### TMA study population

The four available TMAs included 763 individual cores accounting for 207 patients with histopathological diagnosis of glioblastoma, each patient case consisting of 1–9 different cores. Of these cores, 336 (unevenly divided over 23 patients (11%)) could not be assessed due to missing or poor condition of the tissue or absence of tumor cells.

Finally, 427 cores unevenly divided among 184 patients (89%) were available for analysis. Six of these patients were represented twice by having cores of both a sample at first diagnosis and a sample of recurrent tumor resulting in 190 tumors. The mean age at time of diagnosis was 59 years (range 21-87 years) and 70 patients (38%) were female.

### CXCR4 staining

**Fig. 3:**
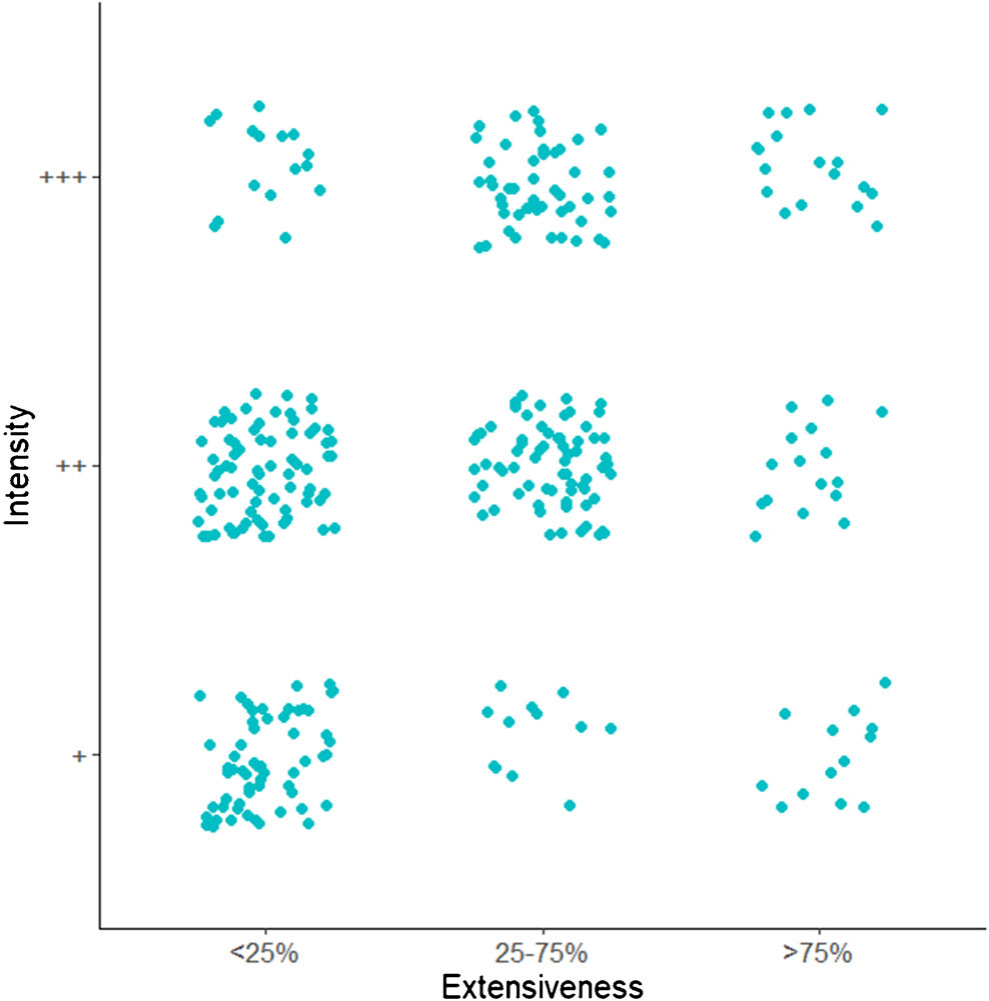
Variation in CXCR4 staining of glioblastoma tissue cores. Every dot represents a CXCR4 positive glioblastoma tissue core with positive CXCR4 staining

Interobserver agreement calculations showed good agreement for tumor cell staining with a Kappa of 0.81, and fair agreement for the presence of stained blood vessels with a Kappa of 0.31. Seventy-eight cores (18%) were scored CXCR4 negative (no staining at all), and 18 cores (4.2%) had both strong staining as well as > 75% staining of the tumor cells. CXCR4 staining in the remaining 331 cores (78%) was highly variable (Fig. 3), also within individual glioblastoma patients. Of all cores with any staining, 255 cores (60%) showed positive cytoplasm of the tumor cells, 250 cores (59%) showed staining of the tumor cell nuclei and 127 cores (30%) showed staining of blood vessels. Fifty cores (12%) showed staining of all three of these tissue components. All four samples of normal brain tissue taken from epileptic surgery cases were CXCR4-negative. Table 1 shows the results for the three cut-offs for dichotomization of CXCR4 staining. The intra-tumor variation found in the cores remains present for all three different cut-offs.

**Table 1:** Dichotomization of CXCR4 staining to define CXCR4 positive, negative, and mixed (e.g., having both CXCR4-positive and negative cores) tumors according to three cut-offs: 1 = positive when at least subtle (+) and limited (< 25%) staining; 2 = positive when at least moderate (++), partial (25-75%) staining; 3 = positive when at least strong (+++), diffuse (>75%) staining

**Cut-off**	*CXCR4* *negative tumor*	*CXCR4* *positive tumor*	*CXCR4* *mixed tumor*
*1 (+ / <25%)*	23 (12%)	142 (75%)	25 (13%)
*2 (++ / 25-75%)*	92 (48%)	49 (26%)	49 (26%)
*3 (+++ / >75%)*	174 (91%)	5 (3%)	11 (6%)

### Tumor markers, survival, and CXCR4 staining

**Fig. 4:**
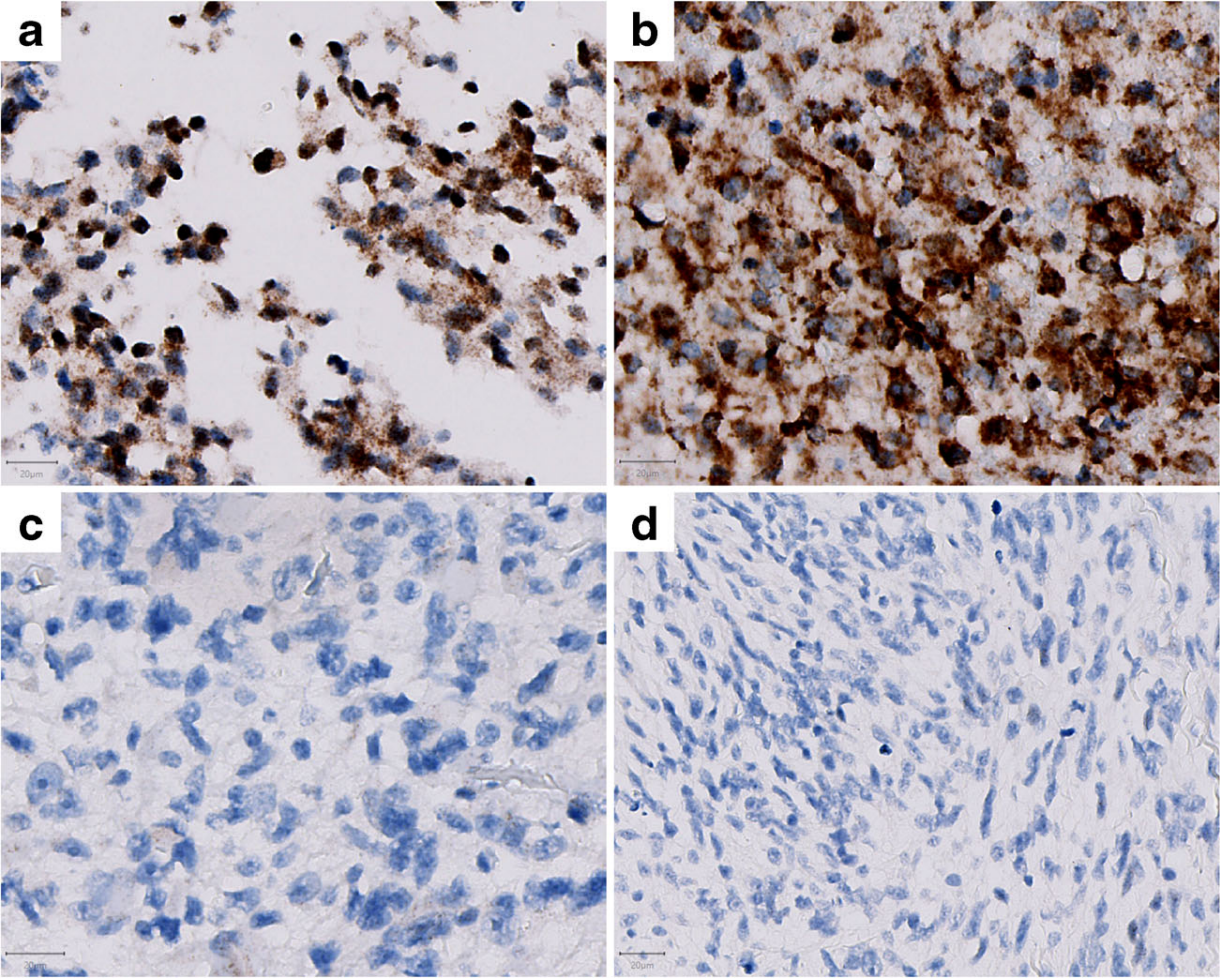
Examples of positive and negative CXCR4 stained glioblastoma tissue cores with different IDH status. The brown color represents staining with the CXCR4 antibody. (**a**) CXCR4 positive IDH mutant glioblastoma tissue of a 62-year-old male with strong CXCR4 staining as well as > 75% staining of the core; (**b**) CXCR4 positive IDH wildtype glioblastoma tissue of a 82-year-old male with strong CXCR4 staining as well as > 75% staining of the core; (**c**) CXCR4 negative IDH mutant glioblastoma tissue of a 54-year-old male and (**d**) CXCR4 negative IDH wildtype glioblastoma tissue of a 59-year-old male. Scale bar is 20 μm

**Fig. 5:**
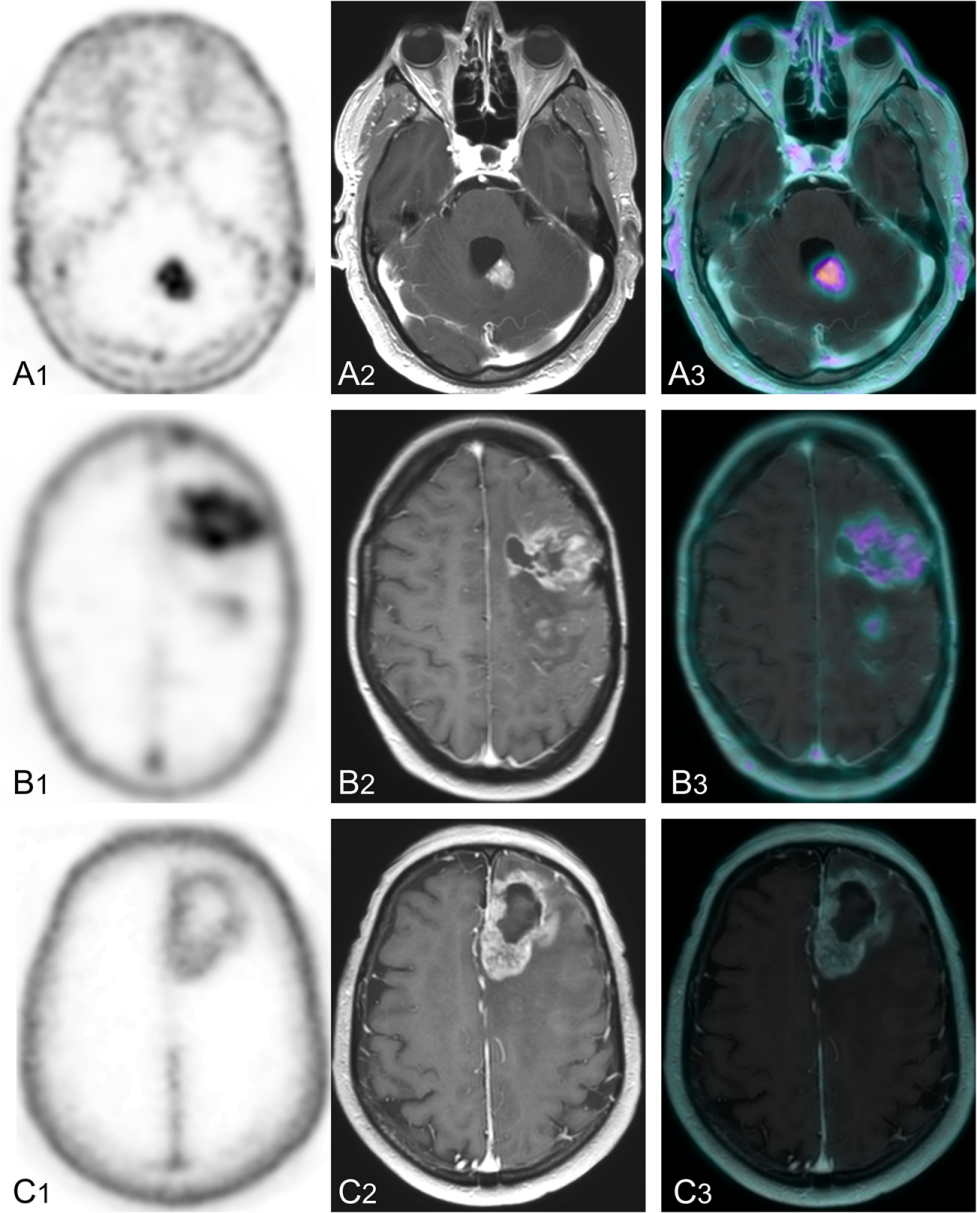
Axial [^68^Ga]Ga-Pentixafor PET (A1; B1; C1), T_2_-weighted MRI (A2; B2; C2) and fused [^68^Ga]Ga-Pentixafor PET/MRI (A3; B3; C3) images of three patients with suspicion of recurrent glioblastoma. (A) Male (patient no. 4 in Table 4) showing higher uptake (SUVmax 3.5) in the MR-enhancing tissue in the left cerebellar hemisphere compared to the other patients and than bloodpool activity (SUVmean 1.48). (B) Female (patient no. 2) showing low to moderate uptake (SUVmax 1.82) in the MR-enhancing tissue in the left frontal lobe slightly higher than bloodpool activity (SUVmean 1.23). (C) Male (patient no. 6) showing low uptake (SUVmax 1.46) in the MR-enhancing tissue in the left frontal lobe equal to bloodpool activity (SUVmean 1.48)

In eleven patients (6.0%) an IDH mutant glioblastoma was found, while 133 patients (72%) had IDH wild-type glioblastoma using immunohistochemical analysis; for the remaining 40 patients (22%), IDH status was unknown. Of the 47 patients (26%), the *MGMT* promoter methylation status was known: 20 of these patients (43%) were *MGMT* methylated. When analyzing the relationship between IDH status respectively *MGMT* promoter methylation and CXCR4 staining scores on a core level, no significant correlations were found (Supplemental Table 1). Similar results were seen on a tumor level for all different cut-off values (Table 2 and Fig. 4). In addition, supportive results were found in the survival analysis, where no significant differences in overall survival were found when comparing the survival distributions of patients with CXCR4 positive, negative or mixed tumors for all three cut-off values (Table 3 and Fig. 5).

**Table 2:** Correlation between CXCR4 staining and molecular tumor markers IDH and *MGMT* on tumor level, according to three cut-offs: 1 = positive when at least subtle (+) and limited (<25%) staining; 2 = positive when at least moderate (++) and partial (25-75%) staining; 3 = positive when at least strong (+++) and diffuse (> 75%) staining; numbers account for tumors

**Cut-off**	*IDH mutant* *(N = 24)*			*IDH* *wildtype* *(N = 316)*			*p value*	*MGMT methylated* *(N = 49)*			*MGMT* *unmethylated* *(N = 79)*			*p value*
	*N*	*P*	*M*	*N*	*P*	*M*		*N*	*P*	*M*	*N*	*P*	*M*	
*1* *(+ / <25%)*	-	10	1	15	101	17	0.614	1	19	-	-	25	2	0.098
*2* *(++ / 25-75%)*	2	6	3	63	34	36	0.261	9	5	6	7	8	12	0.186
*3* *(+++ / >75%)*	9	-	2	121	3	9	0.230	20	-	-	25	-	2	0.500

N = CXCR4 negative tumor; P = CXCR4 positive tumor; M = CXCR4 mixed tumor

**Table 3:** Correlation between overall survival in months and CXCR4 staining on tumor level, according to three cut-offs: 1 = positive when at least subtle (+) and limited (<25%) staining; 2 = positive when at least moderate (++) and partial (25–75%) staining; 3 = positive when at least strong (+++) and diffuse (> 75%) staining; numbers account for months

**Cut-off**	*Mean survival*			*Median survival*			*p value (log rank)*
	*N*	*P*	*M*	*N*	*P*	*M*	
*1(+ / <25%)*	21	21	29	11	14	13	0.491
*2* *(++ / 25-75%)*	23	20	22	14	15	13	0.947
*3(+++ / >75%)*	22	23	22	14	10	13	0.950

N = CXCR4 negative tumor; P = CXCR4 positive tumor; M = CXCR4 mixed tumor

### In vivo-ex vivo correlation

Between November 2019 and August 2020, seven patients (aged 21–75 years at initial diagnosis; two females) with suspicion of recurrent glioblastoma received [^68^Ga]Ga-Pentixafor PET. Details of these patients can be found in Table 4; representative examples of [^68^Ga]Ga-Pentixafor PET images in Fig 5. All patients showed low to moderate uptake in the tumor (mean SUVmean 1.45; mean SUVmax 2.06) compared with bloodpool activity (mean SUVmean 1.36). The very low background activity (mean SUVmean 0.03; mean SUVmax 0.10) leads to a relatively high TBR (mean TBRmean 67.0; mean TBRmax 65.6) even with low to moderate uptake.

**Table 4:** In vivo-ex vivo correlation of seven patients with suspicion of recurrent glioblastoma that received [^68^Ga]Ga-Pentixafor PET. CXCR4 staining is scored for intensity of tumor cells throughout the section as well as the presence of stained tumor vessels.

Patient	Age at initial diagnosis	Sex	IDH status	Tumor		Bloodpool	Background		TBRmax	TBRmean	CXCR4 staining		Time between scan and pathology
				**SUVmax**	**SUVmean**	**SUVmean**	**SUVmax**	**SUVmean**			**Tumor cells**	**Tumor vessels**	
1	47	M	mt	2.6	1.75	1.36	0.08	0.02	130	87.5	Negative to strong areas	+	5 m
2	21	F	mt	1.82	1.05	1.23	0.18	0.06	30.3	17.5	Negative to subtle/moderate areas	+	3 d
3	26	M	wt	0.98	0.79	1.44	0.15	0.04	24.5	19.8	Negative to strong areas	+	5 m
4	56	M	wt	3.5	2.61	1.48	0.08	0.02	175.0	130.5	Negative to moderate/strong areas	+	8 m
5	63	F	wt	2.96	2.06	1.01	0.89	0.69	35.0	29.0	Negative	-	8 m
6	75	M	wt	1.46	1.17	1.48	0.04	0.01	146.0	117.0	Negative to strong areas	+	1 w
7	55	M	wt	2.32	1.35	1.35	0.04	0.02	116.0	67.5	Negative to subtle/ moderate areas	-	8 m

d = day; F = female; m =month; M = male; mt = mutant; w = week; wt = wildtype; + = present; - = absent

**Fig. 6:**
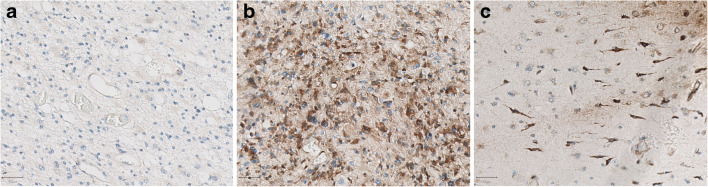
Example of intra-tumoral heterogeneity of CXCR4 staining in recurrent glioblastoma tissue of patient no. 6. (**a**) No staining. (**b**) Extensive and partly strong cytoplasmic staining of tumor and microvascular cells. (**c**) Strong cytoplasmic staining of small subset of neurons (with the appearance of “dark neurons”) in tumor-infiltrated cortex. Scale bar is 50 μm

Comparable to the TMA analysis, all patients showed large intra-tumor variation for CXCR4 staining, even within one section. However, this variation did not fully correspond with the variation of [^68^Ga]Ga-Pentixafor uptake; for instance, patient no. 3 showed low uptake, but CXCR4 tumor cell staining varying from negative to strong. Only one patient (no. 4) showed higher tumor uptake compared with the other patients that indeed corresponded with locally moderate to strong CXCR4 tumor cell staining; unfortunately, due to a combination of factors there was not enough clinical support to proceed to therapy with [^177^Lu]Lu-Pentixather. In addition, another patient (no. 5) was CXCR4 negative with a corresponding lower [^68^Ga]Ga-Pentixafor uptake (Table 4). Interestingly, in one patient (no. 6), a small subset of neurons (with the appearance of “dark neurons”) in tumor-infiltrated cortex also showed strong cytoplasmic CXCR4 staining, while the vast majority of neurons was completely negative (Fig. 6).

## Discussion

In the current study, we assessed the degree of CXCR4 expression in glioblastoma tissue and its possible correlation with molecular tumor markers and survival, to evaluate the potential of CXCR4 as a PET target for the diagnostic PET agent [^68^Ga]Ga-Pentixafor and its therapeutic variant [^177^Lu]Lu-Pentixather in glioblastoma patients. Our most striking finding was the large inter-and intra-tumor variation of CXCR4 expression in glioblastoma tissue, both in terms of degree of expression—as measured by intensity and extensiveness respectively mRNA expression—as well as the different types of tissue components that showed expression. Furthermore, a substantial proportion of cores and tumors did not show any expression.

Our findings are in contrast with some other immunohistochemical studies reporting that all glioblastomas are CXCR4 positive: Komatani et al. presented 24/24 CXCR4 positive glioblastomas and Stevenson et al. 5/5 CXCR4 positive glioblastomas [17, 27]. However, another study showed similar results to ours: Bian et al. studied 44 high-grade gliomas of which six turned out to be CXCR4 negative and 38 CXCR4 positive [14]. To our knowledge, our study is the largest immunohistochemical study to assess CXCR4 expression in glioblastoma tissue, including separate scores for intensity and extensiveness, which might have facilitated the exposure of this large variation in CXCR4 expression. These other studies only reported dichotomized CXCR4 expression. Moreover, different antibodies have been used throughout these studies, possibly leading to different results.

We did not find a significant correlation between CXCR4 expression on the one hand and *IDH* status or *MGMT* promoter methylation on the other, not by immunohistochemistry on a core and tumor basis, nor in the mRNA datasets. Bianco et al. did describe lower expression of CXCR4 in *IDH* mutant glioblastomas; however, they only studied 86 glioblastoma tissue samples, less than half the size of our ex vivo glioblastoma series. [28] Also, no significant differences in survival distributions of CXCR4 positive, negative or mixed tumors were found for three different immunohistochemical cut-offs or in the mRNA datasets. This is in contrast to some literature finding significant differences in survival, which could be due to a smaller sample size [14, 16]. Ma et al. also performed mRNA analysis with R2 genomics platform on the same datasets, but opted for a cut-off for CXCR4 expression while we analyzed continous CXCR4 expression [16].

In our study, healthy brain tissue did not show any CXCR4 staining or mRNA expression of CXCR4. Similarly, Bian et al. and Rempel et al. both examined five normal brain tissue samples that were all CXCR4 negative and Yi et al. presented twelve normal brain tissue samples all with “negligible” CXCR4 staining [11, 14, 29]. One glioblastoma core in our ex vivo data showed CXCR4 staining of neurons, and the same was seen in a small subset of neurons in tumor-infiltrated cortex of one of the clinical patients. This finding may support the hypothesis that CXCR4 (like many other receptors) can be upregulated in neurons adjacent to tumor cells.

Taking into account the variable expression of CXCR4 in glioblastoma cells, it seems CXCR4 expression is high in only a subset of glioblastomas. In clinical practice, this would theoretically mean that if a newly discovered tumor expresses CXCR4, [^68^Ga]Ga-Pentixafor PET could be complementary to tumor imaging, and facilitating surgical and radiotherapy planning. In addition, when pathological examination of the tissue after surgery indeed shows high expression of CXCR4, the residual (or possibly recurrent) tumor might be receptive to treatment with [^177^Lu]Lu-Pentixather. This is especially dependent also on the tumor retention of [^177^Lu]Lu-Pentixather or in some cases may be the high energy beta-emitting [^90^Y]Y-Pentixather. However, when no uptake of the agent is seen, this does not mean no tumor tissue is present; it does imply that the patient most likely will not benefit from radionuclide therapy.

Nevertheless, our in vivo-ex vivo correlation results, albeit performed in seven patients only, suggest that translation of our (and others) database and ex vivo findings to clinical practice should be done with caution. Five out of seven patients showed low to moderate uptake, while CXCR4 staining differed from negative to strong. Differences in structures between the CXCR4 antibody used for staining and [^68^Ga]Ga-Pentixafor could be a possible explanation. The study by Lapa et al. also found similar discrepancies between uptake and immunohistochemistry relating them to possible receptor kinetics and internalization. [20] Another explanation in our study could be that the average time between [^68^Ga]Ga-Pentixafor PET scans and pathological examinations for five out of seven patients was relatively long, possibly leading to discrepancies between uptake and immunohistochemistry. Finally, variability in blood-brain barrier (BBB) crossing between patients may form an additional source of variance in [^68^Ga]Ga-Pentixafor uptake.

In the last decade, many PET agents—like [^11^C]C-MET and [^18^F]F-FDG—have been tested for their diagnostic accuracy and value in follow-up monitoring of gliomas. However, apart from one patient that also underwent a FET PET/CT examination, none of the other patients were imaged with one of these more commonly used tracers, limiting a comparison of diagnostic value. Also, the small sample sizes of the current study and single previous study on [^68^Ga]Ga-Pentixafor by Lapa et al. currently preclude any significant comparisons with the more commonly used PET tracers.

This study has several limitations. First, CXCR4 expression was assessed using a qualitative and semiquantitative scoring system, which is inherently sensitive to observer bias. However, interobserver analysis showed good agreement for the scoring of CXCR4 expression of tumor cell staining. Second, the degree of expression of CXCR4 was scored for tumor cells instead of each tissue component individually, i.e., blood vessels, cytoplasm, and tumor cell nuclei. Several studies have reported the presence of CXCR4 on blood vessels as well as on tumor cells surrounding these vessels [6, 7, 11, 15, 18, 28, 30]. In the light of the significant role, CXCR4 plays in angiogenesis, and it would have been interesting to correlate the degree of CXCR4 expression on blood vessels with our molecular tumor markers. However, intensity and extensiveness of CXCR4 staining on such a sub-tissue level would have led to new challenges and observer bias, as difficulty in scoring of the presence of blood vessels was already shown by only fair interobserver agreement. Third, cut-off values for the correlation analyses on a tumor level were arbitrarily defined and could be subject to discussion; for example, it can be debated whether a core with < 25% staining but a very high intensity score should be defined as “negative” based on a cut-off of “25–75%.” However, it is difficult if not virtually impossible to define tumors in a clinically relevant manner without introducing some sort of cut-off values. Finally, a substantial amount of data on molecular tumor markers was missing, resulting in lower statistical power.

In conclusion, although CXCR4 mRNA expression appears to be highest in glioblastomas, glioblastoma tissue itself shows a large variation of CXCR4 expression, both within and between tumors. No correlation between CXCR4 (mRNA) expression and IDH status or *MGMT* promoter methylation nor survival was found within our series. When high CXCR4 expression is present, targeted imaging with [^68^Ga]Ga-Pentixafor might complement tumor imaging while targeted radionuclide therapy could be possible using [^177^Lu]Lu-Pentixather. Especially in an era when curative treatment of glioblastoma does not exist, visualization and targeted radionuclide therapy might improve survival rates of a subset of patients showing CXCR4 expression. Nevertheless, our seven patient examples show caution should be exercised in directly translating ex vivo CXCR4 expression to PET agent uptake, and future studies could be focused on this correlation between CXCR4 staining and uptake of respectively [^68^Ga]Ga-Pentixafor and [^177^Lu]Lu-Pentixather.

## Supplementary Information

ESM 1 (DOCX 13 kb)

## Data Availability

The datasets generated and/or analyzed during the current study are not publicly available as they contain information that could compromise patients’ privacy but are available from the corresponding author on reasonable request and in anonymous form.
