# Supplementary material for: CXCR4 expression in glioblastoma tissue and the potential for PET imaging and treatment with [68Ga]Ga-Pentixafor /[177Lu]Lu-Pentixather
Source: Eur J Nucl Med Mol Imaging. 2021 Feb 7;49(2):481–91. doi: 10.1007/s00259-021-05196-4 (PMC8803771; doi:10.1007/s00259-021-05196-4)
Supplement: Supplementary file 1 — (DOCX 13 kb) [file 259_2021_5196_MOESM1_ESM.docx]

**Supplemental Table 1** Correlation between CXCR4 staining measured by intensity (divided into no staining (-), subtle staining (+), moderate staining (++) and strong staining (+++)) and extensiveness (defined as an estimated percentage of the area of the core showing CXCR4 staining; divided into 0% (negative), less than 25% (limited), 25 up to 75% (partial) and 75% or higher (diffuse)) and molecular tumor markers IDH and *MGMT* on core level; numbers account for cores.

| **Intensity** | *IDH mutant*  *(N = 24)* | *IDH wildtype*  *(N = 316)* | *p value* | *MGMT methylated*  *(N = 49)* | *MGMT*  *unmethylated*  *(N = 79)* | *p value* |
| --- | --- | --- | --- | --- | --- | --- |
| - | 3 | 53 | 0.052 | 1 | 3 | 0.788 |
| + | 3 | 67 |  | 6 | 19 |  |
| ++ | 8 | 137 |  | 36 | 38 |  |
| +++ | 10 | 59 |  | 6 | 19 |  |
| **Extensiveness** |  | | | | | |
| 0% | 3 | 53 | 0.098 | 1 | 3 | 0.098 |
| <25% | 6 | 137 |  | 24 | 37 |  |
| 25-75% | 12 | 97 |  | 21 | 33 |  |
| ≥ 75% | 3 | 29 |  | 3 | 6 |  |
